# Dysphagia in patients with severe COVID-19: a retrospective study

**DOI:** 10.1038/s41598-024-57508-x

**Published:** 2024-03-21

**Authors:** Rie Asayama, Kaori Tanaka-Nishikubo, Masahiro Okada, Naoki Mukai, Suguru Annen, Hironori Matsumoto, Jun Takeba, Norio Sato, Naohito Hato

**Affiliations:** 1 Department of Otolaryngology, Head and Neck Surgery, Ehime University Graduate School of Medicine, Shitsukawa, Toon, Ehime 791-0295 Japan; 2 Department of Emergency and Critical Care Medicine, Ehime University Graduate School of Medicine, Shitsukawa, Toon, Ehime 791-0295 Japan

**Keywords:** SARS-CoV-2, Swallowing function, Dysphagia, Intubation, Tracheostomy, Diseases, Risk factors

## Abstract

To investigate dysphagia after extubation in patients with severe coronavirus disease 2019 (COVID-19). We retrospectively examined patients with severe COVID-19 treated in our hospital between August 2021 and March 2022. Feeding outcomes were categorized into two groups—(1) total oral intake, and (2) difficulty in oral intake. To assess the feeding outcome, we used modified water-swallowing test (MWST) for all patients. However, in cases where aspiration or recurrent laryngeal nerve palsy was suspected, we conducted the fiberoptic endoscopic evaluation of swallowing after MWST. Patient data were collected from medical records. Forty-six patients with severe COVID-19 were included. Among the 46 patients, 14 (30.4%) experienced difficulties with oral intake. Older age, longer length of hospitalization, duration of mechanical ventilation, tracheostomy, diabetes, and higher serum levels of C-reactive protein (CRP) and procalcitonin (PCT) at the time of intubation were associated with difficulty in oral intake. The rate of difficulty with oral intake in patients with severe COVID-19 was 30.4%, which is not as high as reported in previous studies. Older age, longer duration of mechanical ventilation, tracheostomy, diabetes, and higher levels of CRP and PCT were associated with the prevalence of oral intake difficulty, suggesting that early attention should be paid to high-risk patients who have preexisting deterioration of swallowing function due to aging and comorbidities, or who have prolonged intubation or tracheostomy to prevent aspiration pneumonia.

## Introduction

The outbreak of coronavirus disease 2019 (COVID-19), caused by severe acute respiratory syndrome coronavirus-2 (SARS-CoV-2), has been increasing worldwide since a case was reported in Wuhan, China, in late December 2019^1^. Its clinical symptoms include fever, fatigue, olfactory disturbances, sore throat, and taste disturbances^2^. COVID-19 causes acute respiratory distress syndrome (ARDS) in approximately 20% of hospitalized patients^3^, and patients with severe ARDS require orotracheal intubation. The management of patients with severe COVID-19 among healthcare workers remains a challenge. Patients with severe COVID-19 frequently develop dysphagia^4^; approximately 30–90% of patients with severe COVID-19 have dysphagia^5–11^. In addition, patients with severe COVID-19 have been reported to be at high risk due to respiratory symptoms and reduced lung function^11^.

However, in patients with airway infections such as COVID-19 infection, the videofluoroscopic swallowing study (VFSS) and fiberoptic endoscopic evaluation of swallowing (FEES), which are the gold standards for the evaluation of swallowing function, cannot be easily performed due to multiple perspectives, such as the risk of infection to healthcare workers and medical resources. Although vaccination against SARS-CoV-2 is associated with the severity of COVID-19^12^, the dietary management of patients with severe COVID-19 remains a problem among healthcare workers. This study aimed to investigate the postextubation feeding outcomes and associated risk factors in Japanese patients with severe COVID-19.

## Materials and methods

This retrospective study was approved by the Institutional Review Board of Ehime University Medical Hospital (No. 2108028). The need for informed consent was waived due to the retrospective design of the study by the Institutional Review Board of Ehime University Medical Hospital. However, we presented an opportunity to decline participation on our website. This study was carried out according to the ethical standards of the Institutional Review Board of the Ehime University Medical Hospital and the Declaration of Helsinki. We could not identify individual participants after date collection.

Patients with severe COVID-19 treated at our hospital between August 2021 and March 2022 were enrolled in this study. Our hospital has more than 600 beds and provides tertiary emergency care. Patients transferred to another hospital before the swallowing function test or oral intake test were excluded.

A comprehensive bedside evaluation, including the modified water-swallowing test (MWST), is usually performed after extubation, and FEES is performed in suspected cases, such as aspiration and recurrent laryngeal nerve palsy after MWST. Oral intake is recommended if there are no signs of aspiration during these evaluations.

In this study, feeding outcome was placed into one of two categories: (1) total oral intake, which was defined as 4 ≤ functional oral intake scale (FOIS)^13^ and (2) difficulty in oral intake, which was defined as 3 ≥ FOIS. “Severe” COVID-19 is defined as the need for intubation and mechanical ventilation.

Patient data, including age, sex, body mass index (BMI), length of hospitalization, duration of mechanical ventilation and tracheostomy, use of extracorporeal membrane oxygenation (ECMO), complications including hypertension, dyslipidemia, diabetes, and serological tests including lactic dehydrogenase (LDH), creatine kinase (CK), C-reactive protein (CRP), and procalcitonin (PCT) at the time of intubation were collected from medical records.

Data are expressed as mean and 95% confidence interval (CI). Each parameter was compared between the two groups using the Mann–Whitney U test or Fisher’s exact test with JMP software for Macintosh (SAS Institute Inc., Cary, NC, USA). Statistical significance was set at *p* < 0.05.

## Results

A total of 67 patients with severe COVID-19 were treated in our hospital between August 2021 and March 2022. Of the 67 patients, 12 died and nine were transferred to another hospital before swallowing function test or oral intake. Finally, 46 patients who underwent swallowing function tests or tolerated oral intake were enrolled in this study. The age ranged from 36 to 82 years, with a median age of 58 ± 1.4 (standard error) years. Swallowing function tests were performed at a median of 2 ± 2.9 (standard error) days after extubation or tracheostomy.

Of the 46 patients, 14 (30.4%) experienced difficulties with oral intake. Table 1 shows that the clinical characteristics depended on the feeding outcome. The age was 68.5 (95% CI 57.8–71.8) years in patients with oral intake difficulty group and 57.0 (95% CI 53.5–62.3) years in patients with oral intake group. Age was significantly higher in the oral intake difficulty group than in the oral intake difficulty group (*p* = 0.03). Sex, BMI, hypertension, and dyslipidemia were not significantly different between the two groups (*p* > 0.99, 0.49, > 0.99, and > 0.99, respectively). The length of hospitalization and duration of mechanical ventilation were significantly longer in the oral intake difficulty group than in the oral intake difficulty group (*p* = 0.007 and < 0.001, respectively). Ten (71.4%) of the 14 patients in the oral intake difficulty group and four (12.5%) of the 32 patients in the oral intake group underwent tracheostomy. Tracheostomy rate was significantly higher in the oral intake difficulty group than in the oral intake group (*p* < 0.001), while the use of ECMO was not significantly different between the two groups (*p* = 0.18). Seven (50.0%) of 14 patients with oral intake had complicated diabetes, while six (18.8%) of 32 patients with oral intake complicated diabetes were significantly different between the groups (*p* = 0.04). Serum levels of LDH and CK at the time of intubation were not significantly different between the groups (*p* = 0.43 and 0.12, respectively), while CRP and PCT levels at the time of intubation were significantly higher in the oral intake difficulty group than in the oral intake group (*p* = 0.01 and 0.02, respectively).

**Table 1:** Comparison of clinical characteristics depends on feeding outcome.

	Oral intake difficulty n = 14 (30.4%)	Total oral intake n = 32 (69.6%)	*p*-value
Age (years) (median, IQR)	68.5 (57.8–71.8)	57.0 (53.5–62.3)	.03
Sex (Female/Male)	4/10	10/22	> .99
BMI (median, IQR)	23.9 (20.6–27.9)	24.7 (23.0–26.4)	.49
Length of hospitalization (days) (median, IQR)	26.0 (20.0–51.0)	13.0 (9.8–18.5)	.007
Duration of mechanical ventilation (days) (median, IQR)	24 (16–45)	9 (7–10)	< .001
Tracheostomy (n, %)	10 (71.4%)	4 (12.5%)	< .001
ECMO (n, %)	4 (28.6%)	3 (9.4%)	.18
Complications			
Hypertension (n, %)	5 (35.7%)	10 (31.3%)	> .99
Dyslipidemia (n, %)	1 (7.1%)	4 (12.5%)	> .99
Diabetes (n, %)	7 (50.0%)	6 (18.8%)	.04
LDH (U/L) (median, IQR)	548.0 (312.8–608.5)	425.0 (319.0–551.0)	.43
CK (U/L) (median, IQR)	253.0 (102.5–416.8)	73.5 (45.8–342.3)	.12
CRP (mg/dL) (median, IQR)	11.8 (7.0–15.6)	5.0 (2.6–8.6)	.01
Procalcitonin (ng/mL) (median, IQR)	0.24 (0.08–2.09)	0.08 (0.04–0.19)	.02

BMI, body mass index; CK, creatine kinase; CI, confidence interval; CRP, C-reactive protein; ECMO, extracorporeal membrane oxygenation.

## Discussion

The rate of difficulty with oral intake in this study was 30.4%. Older age, longer length of hospitalization, longer duration of mechanical ventilation, tracheostomy, diabetes, and higher levels of CRP and PCT were associated with difficulty in oral intake in patients with severe COVID-19.

The rate of dysphagia in patients with severe COVID-19 has been reported to be approximately 30–90%^5–11^. The prevalence of difficulty with oral intake in this study was not as high as reported previously. Differences between our results and those of previous reports may arise from differences in inclusion criteria, definition of feeding outcome, or dysphagia. Since the number of patients with severe COVID-19 was high during this period, we were unable to assess swallowing function in all patients. Nine patients in relatively good general condition were transferred to other hospitals before swallowing function tests or oral intake. Therefore, the actual rate of dysphagia may have been low.

In this study, older age, longer length of hospitalization, longer duration of mechanical ventilation, tracheostomy, and diabetes were associated with the prevalence of difficulty in oral intake. These results are consistent with previous reports^5–11^. Patients with severe COVID-19 are prone to dysphagia due to muscle weakness caused by prolonged intubation^9^, cerebrovascular events, encephalomyelitis, encephalopathy, peripheral neuropathy, and myositis^14^. The mechanisms of postintubation dysphagia in COVID-19 patients include impaired swallowing dysfunction resulting from intensive care, endotracheal intubation, or tracheostomy, as well as swallowing dysfunction due to the pulmonary dysfunction characteristic of COVID-19. Macht et al. identified the potential mechanisms of dysphagia in the ICU, including oropharyngeal and laryngeal trauma, neuromuscular weakness, reduced laryngeal sensitivity, altered senses, gastroesophageal reflux, and impaired synchronization of breathing and swallowing.^15^ The decrease in swallowing function due to the tracheotomy has been indicated as a decrease in sensory input due to disruption of the upper airway, decreased subglottic air pressure, and disuse atrophy of the laryngeal structures^16–18^.

A characteristic complication of COVID-19 is reduced lung function due to shortened and weakened breathing; some patients may develop pulmonary fibrosis. In particular, the course has been observed to be more severe in elderly men with complications^19,20^. Breathing and swallowing are highly emphasized movements^21^. Respiratory pauses during swallowing prevent postswallowing bolus aspiration^22^, and disruption of respiratory rhythm due to tachypnea is associated with dysphagia^22^.

Swallowing function generally deteriorates with age due to age-related changes^23^ or underlying diseases. Therefore, clinicians should be aware of the underlying dysphagia, especially in older patients with severe COVID-19. Longer durations of mechanical ventilation and tracheostomy might have a negative impact on swallowing function.

The use of ECMO and serum levels of LDH and CK were not associated with the prevalence of difficulty in oral intake in this study. ECMO is used when ARDS is difficult to treat with invasive mechanical ventilation. Higher levels of LDH have been associated with a higher risk of poor outcomes in COVID-19 patients, reflecting a severe form of interstitial pneumonia (IP)^24^. COVID-19 has also been reported to be associated with viral myositis attributed to direct myocyte invasion or the induction of autoimmunity, and enzyme markers, such as CK, are elevated^25^. Therefore, the use of ECMO and higher levels of LDH and CK are associated with the severity of COVID-19, reflecting ARDS, IP, and myositis. The results of this study suggest that the severity of ARDS, IP, and myositis is not associated with the prevalence of dysphagia. Several previous reports have shown that the severity of the disease is associated with dysphagia^9,26^; however, our results were inconsistent with these previous reports. This may be related to differences in the definition of dysphagia. However, more studies are required to confirm these findings.

Higher levels of CRP and PCT at the time of intubation were significantly associated with difficulty in oral intake, suggesting that patients with bacterial infections or aspiration pneumonia before intubation tend to have a longer duration of mechanical ventilation and dysphagia. Dysphagia has also been reported to be prevalent even in non-intubated patients with COVID-19^27,28^. Together with our results, these findings suggest that patients with higher levels of CRP and PCT may have dysphagia and aspiration pneumonia.

In this study, older age, longer length of hospitalization, longer duration of mechanical ventilation, tracheostomy, diabetes, and higher levels of CRP and PCT at intubation were associated with dysphagia in patients with severe COVID-19. Postoperative oral intake should be performed with caution in patients with these risks. It is difficult to perform swallowing function tests, including FEES, in all cases of severe COVID-19 due to the risk of infection for healthcare workers. Swallowing function tests are recommended for patients with these risk factors.

This study has several limitations. First, this study was retrospective. Because the number of patients was small, we were unable to perform other statistical tests, such as multivariate regression. However, a large-scale prospective study is required to confirm these findings. Second, we performed only the MWST or FEES and did not perform other tests, such as VFSS or high-resolution manometry. Therefore, the detailed mechanisms underlying dysphagia remain unknown. More studies are required, including other swallowing function tests, to confirm the mechanism of dysphagia caused by severe COVID-19. Third, controls other than those with COVID-19 were excluded from this study. We were unable to assess the influence of laryngeal inflammation caused by SARS-CoV-2 infection. More studies that include a control group, are required. Fourth, there was a risk of bias, as we could not assess swallowing function in all patients with severe COVID-19. Due to the high number of patients with severe COVID-19 during this period, patients in relatively good general condition were transferred to other hospitals before swallowing function test or oral intake. Therefore, the incidence of dysphagia may have been low.

## Conclusion

The incidence of difficulty in oral intake in patients with severe COVID-19 was 30.4% which is not as high as reported in previous studies. Older age, longer length of hospitalization, longer duration of mechanical ventilation, tracheostomy, diabetes, and higher levels of CRP and PCT were associated with oral intake difficulty in patients with severe COVID-19. It suggests that early attention should be paid to high-risk patients who have preexisting deterioration of swallowing function due to aging and comorbidities, or who have prolonged intubation or tracheostomy to prevent aspiration pneumonia.

## Data Availability

The datasets generated and/or analyzed during the current study are available from the corresponding author on reasonable request.
